# Tetra­aqua­bis­(6-chloro­pyridine-3-carboxyl­ato-κ*O*)nickel(II) tetra­hydrate

**DOI:** 10.1107/S160053681204322X

**Published:** 2012-10-20

**Authors:** Qiao-Hua Xia, Zhong-Fu Guo, Li Liu, Jian-Quan Lv, Bing Li

**Affiliations:** a College of Sciences, Zhejiang A&F University, Lin’an, Hangzhou, Zhejiang 311300, People’s Republic of China

## Abstract

In the title compound, [Ni(C_6_H_3_ClNO_2_)_2_(H_2_O)_4_]·4H_2_O, the Ni^II^ ion is located on an inversion centre and is octa­hedrally coordinated by four O atoms from four water mol­ecules in the equatorial plane and two O atoms of two 6-chloro-3-carboxyl­ate ligands in axial positions. There are also four lattice water molecules present. The organic ligands are bound to the Ni^II^ ion in a monodentate manner through a carboxyl­ate O atom. There is one strong intra­molecular O—H⋯O hydrogen bond and six inter­molecular O—H⋯O and O—H⋯N hydrogen-bonding inter­actions in the packing, resulting in a complex three-dimensional network structure.

## Related literature

For background to complexes based on 6-chloro­nicotinic acid, see: Long *et al.* (2007 ▶); Li *et al.* (2006 ▶).
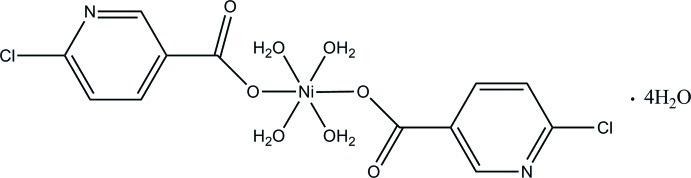


## Experimental

### 

#### Crystal data

[Ni(C_6_H_3_ClNO_2_)_2_(H_2_O)_4_]·4H_2_O
*M* *_r_* = 515.91
Triclinic, 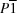

*a* = 7.0245 (14) Å
*b* = 7.3436 (15) Å
*c* = 11.547 (2) Å
α = 86.35 (3)°
β = 77.78 (3)°
γ = 64.55 (3)°
*V* = 525.4 (2) Å^3^
*Z* = 1
Mo *K*α radiation
μ = 1.24 mm^−1^
*T* = 293 K
0.39 × 0.29 × 0.16 mm

#### Data collection

Rigaku R-AXIS RAPID diffractometer
Absorption correction: multi-scan (*ABSCOR*; Higashi, 1995 ▶) *T*_min_ = 0.754, *T*_max_ = 0.862
5167 measured reflections
2371 independent reflections
2170 reflections with *I* > 2σ(*I*)
*R*_int_ = 0.049

#### Refinement

*R*[*F* ^2^ > 2σ(*F* ^2^)] = 0.028
*wR*(*F* ^2^) = 0.076
*S* = 1.06
2371 reflections
134 parameters
H-atom parameters constrained
Δρ_max_ = 0.47 e Å^−3^
Δρ_min_ = −0.41 e Å^−3^

### 

Data collection: *RAPID-AUTO* (Rigaku, 1998 ▶); cell refinement: *RAPID-AUTO*; data reduction: *CrystalStructure* (Rigaku/MSC, 2004 ▶); program(s) used to solve structure: *SHELXS97* (Sheldrick, 2008 ▶); program(s) used to refine structure: *SHELXL97* (Sheldrick, 2008 ▶); molecular graphics: *SHELXTL* (Sheldrick, 2008 ▶); software used to prepare material for publication: *SHELXL97*.

## Supplementary Material

Crystal structure: contains datablock(s) global, I. DOI: 10.1107/S160053681204322X/bq2376sup1.cif

Structure factors: contains datablock(s) I. DOI: 10.1107/S160053681204322X/bq2376Isup2.hkl

Additional supplementary materials:  crystallographic information; 3D view; checkCIF report

## Figures and Tables

**Table 1:** Hydrogen-bond geometry (Å, °)

*D*—H⋯*A*	*D*—H	H⋯*A*	*D*⋯*A*	*D*—H⋯*A*
O*W*2—H2*WA*⋯O2^i^	0.83	1.83	2.6401 (18)	167
O*W*1—H1*WB*⋯O*W*3^ii^	0.81	2.07	2.869 (2)	167
O*W*1—H1*WA*⋯O*W*4^iii^	0.82	1.97	2.7915 (19)	179
O*W*4—H4*WA*⋯N^iv^	0.81	2.14	2.850 (2)	147
O*W*3—H3*WB*⋯O*W*4^v^	0.80	1.99	2.763 (2)	163
O*W*3—H3*WA*⋯O*W*2^vi^	0.81	2.21	2.933 (2)	149
O*W*2—H2*WB*⋯O*W*3	0.84	1.98	2.819 (2)	177
O*W*4—H4*WB*⋯O2	0.82	1.97	2.764 (2)	162

Symmetry codes: (i) 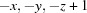; (ii) 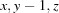; (iii) 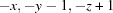; (iv) 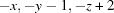; (v) 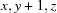; (vi) 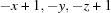.

**Fig. 1.**
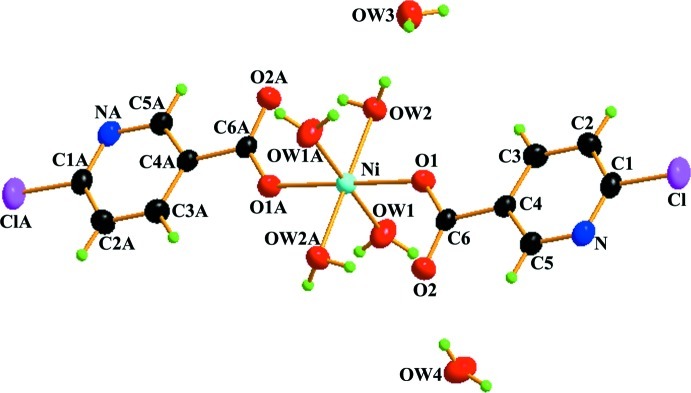
The molecular structure of the title complex, with 50% probability displacement ellipsoids for non-H atoms. Symmetry codes: (A) -x, -y, 1 - z.

**Fig. 2.**
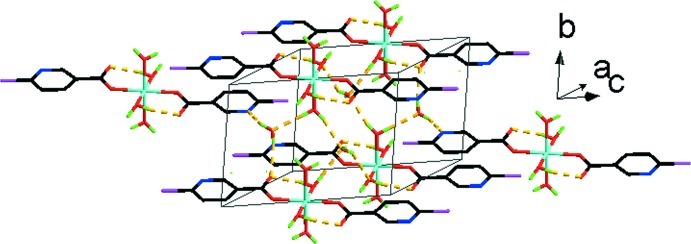
Crystal packing diagram for the title compound. All atoms are shown as isotropic spheres of arbitrary size. H atoms bonded to C atoms are omitted for clarity. The H-bonding interactions are shown as yellow dashed lines.

## supplementary crystallographic information

### Comment

The asymmetric unit of the title complex, contains a half of Ni^II^ ion lying on a crystallographic inversion center, 6-chloropyridine-3-carboxylate anion, two coordinated water molecules and two lattice water molecules (Fig. 1). The Ni^II^ ion is six-coordinated within a octahedral, NiO_6_ coordination geometry. Six coordination arises from two 6-chloropyridine-3-carboxylate ligands in the apical positions [Ni—O = 2.0335 (12) Å] and four water molecules in the equatorial plane [Ni—O = 2.0742 (16) Å and 2.0814 (12) Å]. The bond angles around the Ni center lie within the range 88.8–91.2° for the formally *cis* pairs of ligating atoms. The 6-chloropyridine-3-carboxylate carboxylate ligands are bound to the Ni^II^ ion in a monodentate mode through a carboxylate O atom. There is one strong intramolecular hydrogen bond of type O—H···O, formed by coordinated water atom OW2 and uncoordinated carboxylate atom O2, as well as six strong intermolecular hydrogen bonds of O—H···O type and one of O—H···N type in the packing of the title compound forming a three-dimensional supramolecular structure (Fig.2).

### Experimental

All commercially obtained reagent grade chemicals were used without further purification. A mixture of nickelous sulfate hexahydrate (0.5698 g) and 6-chloronicotinic acid (0.1701 g) were added into 20 ml water and with 20 drops of 0.1 mol/*L* sodium hydroxide solution, and then stirred for 1 h. Finally, 10 ml 95% ethanol carefully layered above-mentioned solution in glass tube. After 1 days, green crystals of the title complex were collected.

### Refinement

H atoms bonded to C atoms were introduced in calculated positions and refined using a riding model [C—H = 0.93 Å and *U*_iso_(H) = 1.2Ueq(C) for aromatic H atoms. H atoms belonging to water molecules were found in difference Fourier maps.

### Crystal data

**Table:**

[Ni(C_6_H_3_ClNO_2_)_2_(H_2_O)_4_]·4H_2_O	*Z* = 1
*M**_r_* = 515.91	*F*(000) = 266
Triclinic, *P*1	*D*_x_ = 1.630 Mg m^−^^3^
Hall symbol: -P 1	Mo *K*α radiation, λ = 0.71073 Å
*a* = 7.0245 (14) Å	Cell parameters from 4651 reflections
*b* = 7.3436 (15) Å	θ = 3.1–27.5°
*c* = 11.547 (2) Å	µ = 1.24 mm^−^^1^
α = 86.35 (3)°	*T* = 293 K
β = 77.78 (3)°	Block, green
γ = 64.55 (3)°	0.39 × 0.29 × 0.16 mm
*V* = 525.4 (2) Å^3^	

### Data collection

**Table:**

Rigaku R-AXIS RAPID diffractometer	2371 independent reflections
Radiation source: fine-focus sealed tube	2170 reflections with *I* > 2σ(*I*)
Graphite monochromator	*R*_int_ = 0.049
ω scans	θ_max_ = 27.5°, θ_min_ = 3.1°
Absorption correction: multi-scan (*ABSCOR*; Higashi, 1995)	*h* = −8→9
*T*_min_ = 0.754, *T*_max_ = 0.862	*k* = −9→9
5167 measured reflections	*l* = −14→14

### Refinement

**Table:**

Refinement on *F*^2^	Secondary atom site location: difference Fourier map
Least-squares matrix: full	Hydrogen site location: inferred from neighbouring sites
*R*[*F*^2^ > 2σ(*F*^2^)] = 0.028	H-atom parameters constrained
*wR*(*F*^2^) = 0.076	*w* = 1/[σ^2^(*F*_o_^2^) + (0.0344*P*)^2^ + 0.1122*P*] where *P* = (*F*_o_^2^ + 2*F*_c_^2^)/3
*S* = 1.06	(Δ/σ)_max_ = 0.001
2371 reflections	Δρ_max_ = 0.47 e Å^−^^3^
134 parameters	Δρ_min_ = −0.41 e Å^−^^3^
0 restraints	Extinction correction: *SHELXL97* (Sheldrick, 2008), Fc^*^=kFc[1+0.001xFc^2^λ^3^/sin(2θ)]^-1/4^
Primary atom site location: structure-invariant direct methods	Extinction coefficient: 0.115 (6)

### Special details

**Table:**

Geometry. All e.s.d.'s (except the e.s.d. in the dihedral angle between two l.s. planes) are estimated using the full covariance matrix. The cell e.s.d.'s are taken into account individually in the estimation of e.s.d.'s in distances, angles and torsion angles; correlations between e.s.d.'s in cell parameters are only used when they are defined by crystal symmetry. An approximate (isotropic) treatment of cell e.s.d.'s is used for estimating e.s.d.'s involving l.s. planes.
Refinement. Refinement of *F*^2^ against ALL reflections. The weighted *R*-factor *wR* and goodness of fit *S* are based on *F*^2^, conventional *R*-factors *R* are based on *F*, with *F* set to zero for negative *F*^2^. The threshold expression of *F*^2^ > σ(*F*^2^) is used only for calculating *R*-factors(gt) *etc*. and is not relevant to the choice of reflections for refinement. *R*-factors based on *F*^2^ are statistically about twice as large as those based on *F*, and *R*- factors based on ALL data will be even larger.

### Fractional atomic coordinates and isotropic or equivalent isotropic displacement parameters (Å^2^)

**Table:**

	*x*	*y*	*z*	*U*_iso_*/*U*_eq_	
Cl	0.49297 (8)	−0.29064 (8)	1.14788 (4)	0.04615 (15)	
Ni	0.0000	0.0000	0.5000	0.02619 (12)	
O1	0.1088 (2)	−0.0624 (2)	0.65419 (10)	0.0345 (3)	
O2	−0.1079 (2)	−0.1909 (2)	0.76064 (10)	0.0369 (3)	
OW1	0.1737 (2)	−0.2994 (2)	0.44232 (11)	0.0438 (3)	
H1WB	0.2196	−0.3961	0.4838	0.066*	
H1WA	0.1310	−0.3310	0.3897	0.066*	
OW2	0.27477 (19)	0.0432 (2)	0.42777 (10)	0.0354 (3)	
H2WB	0.2884	0.1261	0.4680	0.053*	
H2WA	0.2366	0.0978	0.3668	0.053*	
OW3	0.3330 (2)	0.3218 (2)	0.55701 (12)	0.0476 (3)	
H3WB	0.2430	0.3539	0.6168	0.071*	
H3WA	0.4486	0.2539	0.5747	0.071*	
OW4	−0.0265 (2)	−0.5912 (2)	0.73619 (11)	0.0475 (3)	
H4WB	−0.0788	−0.4682	0.7463	0.071*	
H4WA	−0.0208	−0.6457	0.7992	0.071*	
N	0.1923 (2)	−0.2769 (2)	1.04439 (11)	0.0334 (3)	
C1	0.3679 (3)	−0.2457 (3)	1.02767 (14)	0.0316 (3)	
C2	0.4551 (3)	−0.1812 (3)	0.92311 (15)	0.0362 (4)	
H2A	0.5800	−0.1626	0.9159	0.043*	
C3	0.3501 (3)	−0.1457 (3)	0.83059 (14)	0.0347 (4)	
H3A	0.4023	−0.1003	0.7593	0.042*	
C4	0.1653 (2)	−0.1777 (2)	0.84378 (13)	0.0273 (3)	
C5	0.0943 (3)	−0.2442 (3)	0.95247 (13)	0.0312 (3)	
H5A	−0.0282	−0.2673	0.9620	0.037*	
C6	0.0457 (2)	−0.1415 (2)	0.74546 (13)	0.0270 (3)	

### Atomic displacement parameters (Å^2^)

**Table:**

	*U*^11^	*U*^22^	*U*^33^	*U*^12^	*U*^13^	*U*^23^
Cl	0.0594 (3)	0.0454 (3)	0.0361 (2)	−0.0180 (2)	−0.0256 (2)	0.00447 (19)
Ni	0.03131 (17)	0.03082 (19)	0.02103 (16)	−0.01728 (13)	−0.00745 (11)	0.00611 (10)
O1	0.0433 (6)	0.0454 (8)	0.0255 (5)	−0.0280 (6)	−0.0117 (5)	0.0114 (5)
O2	0.0415 (6)	0.0468 (8)	0.0335 (6)	−0.0283 (6)	−0.0127 (5)	0.0120 (5)
OW1	0.0630 (8)	0.0321 (7)	0.0362 (6)	−0.0166 (6)	−0.0186 (6)	0.0031 (5)
OW2	0.0389 (6)	0.0457 (8)	0.0303 (5)	−0.0251 (6)	−0.0116 (5)	0.0085 (5)
OW3	0.0432 (7)	0.0526 (9)	0.0462 (7)	−0.0186 (7)	−0.0140 (6)	0.0092 (6)
OW4	0.0700 (9)	0.0437 (9)	0.0328 (6)	−0.0308 (7)	−0.0047 (6)	0.0033 (6)
N	0.0412 (8)	0.0352 (9)	0.0235 (6)	−0.0162 (6)	−0.0076 (6)	0.0065 (5)
C1	0.0383 (8)	0.0279 (9)	0.0266 (7)	−0.0102 (7)	−0.0114 (6)	0.0018 (6)
C2	0.0357 (8)	0.0462 (11)	0.0326 (8)	−0.0224 (8)	−0.0093 (7)	0.0054 (7)
C3	0.0387 (9)	0.0428 (11)	0.0264 (7)	−0.0224 (8)	−0.0052 (7)	0.0061 (7)
C4	0.0313 (7)	0.0262 (8)	0.0241 (7)	−0.0125 (6)	−0.0053 (6)	0.0036 (6)
C5	0.0347 (8)	0.0342 (10)	0.0263 (7)	−0.0166 (7)	−0.0067 (6)	0.0062 (6)
C6	0.0330 (8)	0.0245 (8)	0.0232 (7)	−0.0119 (6)	−0.0066 (6)	0.0037 (6)

### Geometric parameters (Å, º)

**Table:**

Cl—C1	1.7369 (16)	OW1—H1WB	0.8140
Ni—O1^i^	2.0335 (12)	OW1—H1WA	0.8174
Ni—O1	2.0335 (12)	C4—C5	1.389 (2)
Ni—OW1	2.0742 (16)	C4—C3	1.392 (2)
Ni—OW1^i^	2.0742 (16)	C2—C3	1.374 (2)
Ni—OW2	2.0814 (12)	C2—H2A	0.9300
Ni—OW2^i^	2.0814 (12)	N—C5	1.339 (2)
O1—C6	1.2607 (18)	C5—H5A	0.9300
C6—O2	1.2550 (19)	C3—H3A	0.9300
C6—C4	1.496 (2)	OW4—H4WB	0.8210
C1—N	1.321 (2)	OW4—H4WA	0.8080
C1—C2	1.386 (2)	OW3—H3WB	0.8000
OW2—H2WB	0.8378	OW3—H3WA	0.8121
OW2—H2WA	0.8252		
			
O1^i^—Ni—O1	180.00 (2)	Ni—OW2—H2WB	112.2
O1^i^—Ni—OW1	88.84 (6)	Ni—OW2—H2WA	97.3
O1—Ni—OW1	91.16 (6)	H2WB—OW2—H2WA	108.5
O1^i^—Ni—OW1^i^	91.16 (6)	Ni—OW1—H1WB	126.3
O1—Ni—OW1^i^	88.84 (6)	Ni—OW1—H1WA	114.0
OW1—Ni—OW1^i^	180.0	H1WB—OW1—H1WA	108.8
O1^i^—Ni—OW2	93.00 (5)	C5—C4—C3	117.46 (14)
O1—Ni—OW2	87.00 (5)	C5—C4—C6	120.79 (14)
OW1—Ni—OW2	87.71 (6)	C3—C4—C6	121.74 (14)
OW1^i^—Ni—OW2	92.29 (6)	C3—C2—C1	117.44 (15)
O1^i^—Ni—OW2^i^	87.00 (5)	C3—C2—H2A	121.3
O1—Ni—OW2^i^	93.00 (5)	C1—C2—H2A	121.3
OW1—Ni—OW2^i^	92.29 (6)	C1—N—C5	116.77 (14)
OW1^i^—Ni—OW2^i^	87.71 (6)	N—C5—C4	123.71 (15)
OW2—Ni—OW2^i^	180.00 (3)	N—C5—H5A	118.1
C6—O1—Ni	128.83 (10)	C4—C5—H5A	118.1
O2—C6—O1	125.72 (14)	C2—C3—C4	119.83 (15)
O2—C6—C4	117.79 (14)	C2—C3—H3A	120.1
O1—C6—C4	116.49 (13)	C4—C3—H3A	120.1
N—C1—C2	124.78 (14)	H4WB—OW4—H4WA	110.3
N—C1—Cl	115.91 (13)	H3WB—OW3—H3WA	107.9
C2—C1—Cl	119.31 (13)		

Symmetry code: (i) −*x*, −*y*, −*z*+1.

### Hydrogen-bond geometry (Å, º)

**Table:**

*D*—H···*A*	*D*—H	H···*A*	*D*···*A*	*D*—H···*A*
O*W*2—H2*WA*···O2^i^	0.83	1.83	2.6401 (18)	167
O*W*1—H1*WB*···O*W*3^ii^	0.81	2.07	2.869 (2)	167
O*W*1—H1*WA*···O*W*4^iii^	0.82	1.97	2.7915 (19)	179
O*W*4—H4*WA*···N^iv^	0.81	2.14	2.850 (2)	147
O*W*3—H3*WB*···O*W*4^v^	0.80	1.99	2.763 (2)	163
O*W*3—H3*WA*···O*W*2^vi^	0.81	2.21	2.933 (2)	149
O*W*2—H2*WB*···O*W*3	0.84	1.98	2.819 (2)	177
O*W*4—H4*WB*···O2	0.82	1.97	2.764 (2)	162

Symmetry codes: (i) −*x*, −*y*, −*z*+1; (ii) *x*, *y*−1, *z*; (iii) −*x*, −*y*−1, −*z*+1; (iv) −*x*, −*y*−1, −*z*+2; (v) *x*, *y*+1, *z*; (vi) −*x*+1, −*y*, −*z*+1.
